# Stress CMR in Known or Suspected CAD: Diagnostic and Prognostic Role

**DOI:** 10.1155/2021/6678029

**Published:** 2021-01-14

**Authors:** Francesca Baessato, Marco Guglielmo, Giuseppe Muscogiuri, Andrea Baggiano, Laura Fusini, Stefano Scafuri, Mario Babbaro, Rocco Mollace, Ada Collevecchio, Andrea I. Guaricci, Gianluca Pontone

**Affiliations:** ^1^Department of Cardiology, San Maurizio Regional Hospital, Bolzano, Italy; ^2^Cardiovascular Imaging Department, Centro Cardiologico Monzino IRCCS, Milan, Italy; ^3^Department of Cardiac, Thoracic, Vascular Sciences and Public Health, University of Padua, Padua, Italy; ^4^Institute of Cardiovascular Disease, Department of Emergency and Organ Transplantation, University Hospital Policlinico of Bari, Bari, Italy

## Abstract

The recently published 2019 guidelines on chronic coronary syndromes (CCS) focus on the need for noninvasive imaging modalities to accurately establish the diagnosis of coronary artery disease (CAD) and assess the risk of clinical scenario occurrence. Appropriate patient management should rely on controlling symptoms, improving prognosis, and guiding each therapeutic strategy as well as monitoring disease progress. Among the noninvasive imaging modalities, cardiovascular magnetic resonance (CMR) has gained broad acceptance in past years due to its unique features in providing a complete assessment of CAD through data on cardiac anatomy and function and myocardial viability, with high spatial and temporal resolution and without ionizing radiation. In detail, evaluation of the presence and extent of myocardial ischemia through stress CMR (S-CMR) has shown a high rule-in power in detecting functionally significant coronary artery stenosis in patients suspected of CCS. Moreover, S-CMR technique may add significant prognostic value, as demonstrated by different studies which have progressively evidenced the valuable power of this multiparametric imaging modality in predicting adverse cardiac events. The latest scientific progress supports a greater expansion of S-CMR with improvement of quantitative myocardial perfusion analysis, myocardial strain, and native mapping within the same examination. Although further study is warranted, these techniques, which are currently mostly restricted to the research field, are likely to become increasingly prevalent in the clinical setting with the scope of increasing accuracy in the selection of patients to be sent to invasive revascularization. This review investigates the diagnostic and prognostic role of S-CMR in the context of CAD, by analysing a strong, long-standing, scientific evidence together with an appraisal of new advanced techniques which may potentially enrich CAD management in the next future.

## 1. Introduction

Coronary artery disease (CAD) is a widespread clinical phenomenon associated with different clinical entities, which involves a large burden on the healthcare system with an increasing need for objective diagnostic tests to both confirm the diagnosis and assess the event risk [1–4]. In 2019, the European Society of Cardiology (ESC) published the guidelines on the diagnosis and management of chronic coronary syndromes (CCS), which represent a relevant step by introducing innovative changes mostly in the diagnostic workup of suspected obstructive CAD. Noninvasive imaging methods, either functional tests or anatomical imaging, represent indispensable tools for appropriate management of patients with known or suspected CAD, by providing adequate detection of the disease, guiding therapy, and predicting outcome. Stress CMR (S-CMR) is a functional imaging test that has been widely recognized in the past few years as an accurate, well-validated, nonionizing technique [5–7]. The possibility of a multiparametric approach in each S-CMR study, from reproducible evaluation of cardiac function and scar detection to an accurate definition of myocardial ischemia in hemodynamically relevant coronary stenosis and microvascular dysfunction, has made S-CMR an appealing noninvasive modality for comprehensive assessment of CAD.

## 2. Clinical Applications and Technical Approach

According to the latest version of the guidelines, the diagnostic approach of CAD should be primarily based on the clinical likelihood of the disease, and the choice for the initial diagnostic test, either noninvasive imaging as a “gatekeeper” to invasive coronary angiography (ICA) or direct ICA, should be based on clinical risk assessment, patient characteristics, local expertise, and test availability [8].

Noninvasive imaging methods, either functional tests, such as S-CMR, or anatomical imaging, such as cardiac computed tomography angiography (CCTA), are recommended in Class Ib as the initial tests for diagnosing CAD in symptomatic patients in whom obstructive CAD cannot be excluded by clinical assessment alone [9]. Of note, each imaging test has a different performance in ruling in or ruling out obstructive CAD, which should also be taken into account in the initial workup.

Functional imaging methods include myocardial perfusion imaging with single-photon emission computed tomography (SPECT), positron emission computed tomography (PET), stress echocardiography, and S-CMR. These modalities, although possibly missing subclinical atherosclerosis, typically have better rule-in power and have shown higher specificity for the detection of hemodynamically significant coronary stenosis than anatomical imaging with CCTA, by leading to fewer referrals for ICA compared with a strategy relying on anatomical imaging or exercise ECG only [10].

S-CMR can detect myocardial ischemia, thus functionally significant CAD, through evaluation of perfusion defects or ischemic wall motion abnormalities (WMA) provoked by exercise or pharmacological stress. S-CMR protocol should be performed according to the latest update of the S-CMR guidelines [11] and briefly implies a rest and stress phase, with final late gadolinium enhancement (LGE) sequences.

Vasodilators are the most commonly used stress agents (adenosine, dipyridamole, and regadenoson) which commonly induce myocardial ischemia through a “steal phenomenon” and loss of autoregulation mechanism, thus leading to perfusion defects [12]. In case of perfusion imaging, a first-pass perfusion technique using a saturation-prepared T1-weighted fast gradient echo sequence is performed at peak myocardial stress during contemporary gadolinium contrast agent injection. If dipyridamole is used, additional cine sequences are exploited due to its longer half-life [13].

**Figure 1:**
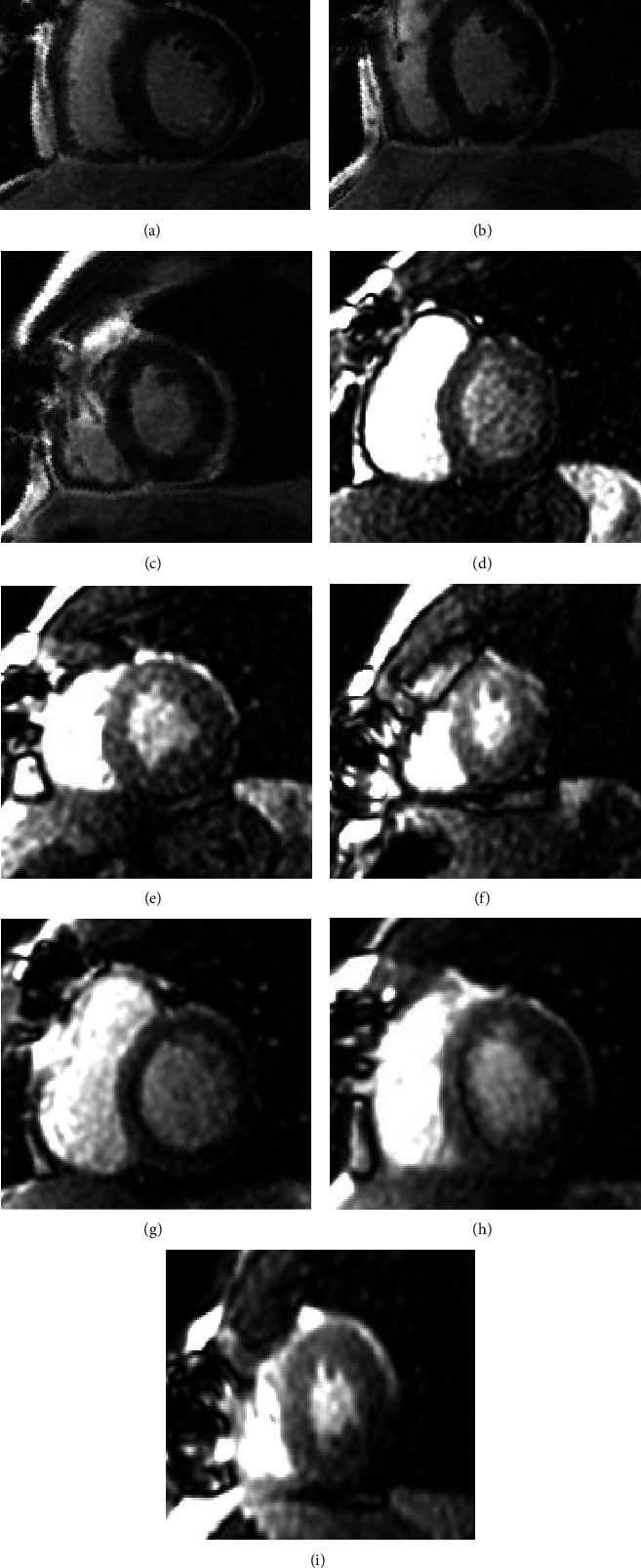
A 60-year-old woman: new-onset angina in previous myocardial infarction and PCI of right coronary artery; LGE sequences (a–c) showed subendocardial inferolateral fibrosis (ischemic pattern); after regadenoson administration, matching rest (d–f) and stress perfusion sequences (g–i) septal reversible perfusion defect was detected. Invasive coronary angiography confirmed subocclusive left descending anterior coronary artery stenosis.

In opposition, inotropic agents, such as dobutamine, act by improving heart rate and only cine sequences are acquired at maximal stress for detection of regional WMA to unmask myocardial ischemia. Hence, each S-CMR examination can be classified as either normal (absence of stress perfusion defect in at least 1 myocardial segment free from LGE) or positive for ischemia (reversible myocardial perfusion defect alone or combined with WMA in at least 1 myocardial segment without corresponding LGE, as shown in Figure 1).

Actually, the recent 2019 guidelines recommend S-CMR (Class Ib) preferentially in patients with higher clinical likelihood of CAD or with a history of revascularization, in whom a functional evaluation of ischemia together with myocardial viability would be most useful, as also supported by cost-effectiveness data [14]. On the other hand, anatomical imaging with CCTA is recommended as first-line test (Class Ib) in suitable patients with low to intermediate clinical likelihood of CAD or no history of CAD, due to its highest rule-out capability [9].

This represents a relevant change compared to the previous version of the guidance, where stress imaging was recommended in patients with stable CAD as the first preferred diagnostic option (Class Ib), while CCTA was given only a Class IIa indication as an alternative test for ruling out significant CAD in selected patients [15].

If recent strong evidences [16, 17] have favoured in Europe a relevant spin-off of CCTA in the field of CCS against functional imaging [18, 19], numerous data have underlined the excellent sensibility and specificity of S-CMR in CAD diagnosis and patient risk classification with a long-standing scientific evidence [20, 21].

## 3. Diagnostic Role of Stress CMR

Numerous studies have reported a high diagnostic accuracy of noninvasive imaging modalities in detecting significant obstructive CAD against clinical gold standards, angiographically determined luminal coronary stenosis and fractional flow reserve (FFR) [22–28].

Concerning S-CMR, there is a wide body of scientific evidence that has strengthened its position, and S-CMR has shown excellent diagnostic performance in the detection of CAD, both for hemodynamically significant coronary stenosis and microvascular dysfunction [29, 30]. Many of these studies regarding the diagnostic performance of S-CMR are listed in Table 1.

In 2001, Schwitter et al. presented one of the first multislice approach studies on perfusion S-CMR in an unselected study population and demonstrated for S-CMR a sensitivity and specificity of 91% and 94%, respectively, for the detection of CAD by S-CMR using PET as gold standard, and a sensitivity and specificity of 87% and 85%, respectively, using quantitative coronary angiography (stenosis > 50%) as gold standard [31]. These results initially sustained the role of perfusion CMR as a reliable modality for the detection of CAD in comparison to other perfusion modalities, with the additional capacity of identifying even subendocardial defects, which are currently missed by SPECT.

A large meta-analysis by Nandalur et al. involving 1183 patients further enhanced the emerging role of S-CMR in the diagnosis of CAD by showing a sensitivity of 91% and a specificity of 81% for perfusion CMR and a sensitivity of 83% and specificity of 86% for stress-induced WMA in a per patient analysis, respectively [32].

Different trials investigated the diagnostic accuracy of S-CMR versus SPECT, a still worldwide used technique that historically has represented the gold standard for myocardial perfusion assessment.

The MR-IMPACT trial in 2008 was a multicentre, multivendor, randomized trial that determined in 241 patients the diagnostic performance of adenosine perfusion-CMR in comparison to SPECT for the detection of CAD, with ICA as the reference standard. Perfusion-CMR at the optimal contrast dose had similar performance as SPECT studies in patients with the same dose (area under ROC curve (AUC): 0.86 ± 0.06 vs. 0.75 ± 0.09 for SPECT, *p* = 0.12), but with even superior diagnostic performance when compared to the entire SPECT population (AUC: 0.67 ± 0.05, *n* = 212; *p* = 0.013). Schwitter et al. were therefore able to demonstrate how S-CMR could at least represent a valuable alternative to SPECT for CAD detection [33].

These evidences were later supported by the larger MR-IMPACT II trial, which involved 533 patients among 33 centres. Patients were evaluated by S-CMR and gated-SPECT before ICA. Both tests showed a nonsignificant difference in terms of percentage of not-evaluable tests (5.6% versus 3.7%, respectively, *p* = 0.21) while S-CMR showed a superior sensitivity in detecting CAD compared to SPECT (sensitivity score of 0.69 and 0.59, respectively, *p* = 0.024), but with a lower specificity (specificity scores of 0.61 and 0.72, respectively, *p* = 0.038) [34]. The overall superiority of S-CMR over SPECT and gated-SPECT for the detection of CAD was demonstrated (AUC 0.75 vs. 0.65 vs. 0.69) with significant difference (*p* = 0.0004 and *p* = 0.018) [35].

Of note, MR-IMPACT trials were performed on a selected population with a relatively high pretest probability of disease, which is not the typical population referred for noninvasive stress tests in clinical practice. However, all tests were performed in all patients to avoid testing bias.

A powerful and direct comparison between S-CMR and SPECT was additionally provided by the CE-MARC study in 2012. This was a large, prospective, multicentre trial that involved a cohort of 628 patients with suspected angina, who prospectively underwent S-CMR, SPECT, and ICA (reference standard) examinations in a period of 4 weeks with later follow-up till 5 years. Of note, the S-CMR examination included a multiparametric protocol with rest and stress (adenosine) perfusion, cine imaging, 3D coronary MR angiography, and LGE. In this study, Greenwood et al. demonstrated a significantly higher sensitivity and negative predictive value of S-CMR compared to SPECT (86% vs. 66%, 90% vs. 79%, respectively, *p* < 0.0001), but with similar specificity and positive predictive values (83% vs. 82%, 77% vs. 71%, respectively, *p* = 0.916 and *p* = 0.061) for detecting significant coronary artery stenosis. Furthermore, S-CMR showed a higher AUC than SPECT (0.89 versus 0.79; *p* < 0.0001) independently of the threshold used to define the presence of obstructive CAD (50% or 70% coronary artery stenose) and regardless of the extension of vessel disease [36].

In detail, a subsequent gender-based subanalysis of the CE-MARC trial showed greater sensitivity of S-CMR than SPECT in both genders, and differently from SPECT, there were no relevant gender differences in the diagnostic accuracy [37].

Different meta-analyses have also evaluated the diagnostic accuracy of S-CMR in identifying CAD by using invasive FFR as reference standard.

In a meta-analysis by Danad et al., S-CMR had the highest performance for the diagnosis of hemodynamically significant CAD on both a per-vessel (AUC 0.97) and per-patient (AUC 0.94) basis, due to excellent sensitivity and specificity. Anatomical evaluation with CCTA and ICA yielded lower specificity, with functional assessment of coronary atherosclerosis by stress echo, SPECT, and FFR-CT improving accuracy [38].

Other data from meta-analyses showed that S-CMR sensitivities and specificities ranged between 89% and 91% and 81% and 86%, respectively [39–41].

Pontone et al. in 2019 compared the diagnostic performance of noninvasive tests using invasive FFR as a reference standard for CAD, including 77 studies. S-CMR showed a higher sensitivity in detecting functionally significant CAD (81%) than stress perfusion CT combined with CCTA (79%), stress perfusion CT (77%), stress echo (72%), and SPECT (64%), despite being inferior to CCTA (88%), FFR-CT (85%), and PET (85%). However, S-CMR showed a higher test performance to identify patients that needed subsequent invasive coronary artery procedures (91%) [42].

Of note, the majority of scientific studies on the diagnostic performance of S-CMR have been performed on adenosine perfusion stress tests, which actually represent the mainstay of S-CMR.

Little scientific evidence exists on other vasodilator agents, such as dipyridamole and regadenoson.

Compared to adenosine, dipyridamole demonstrated reduced sensitivity (86% versus 90%; *p* = 0.022) and similar specificity (77% versus 81%; *p* = 0.065) for diagnosing coronary stenosis ≥ 50% on ICA, but provided additional information by evaluating both perfusion and wall motion abnormalities [43].

Regadenoson and adenosine achieved equivalent vasodilator stress and myocardial perfusion reserve (MPR), but the latter is cheaper and better tolerated [44, 45].

Regarding dobutamine as a stress agent, although it is the only technique that has offered a comparative performance against dobutamine stress echocardiography [46], it is still less used than vasodilator stressors that allow a simpler and safer vasodilatory myocardial perfusion. In a meta-analysis of 37 studies involving 2191 patients, dobutamine-induced RWMA demonstrated a sensitivity of 0.83 and a specificity of 0.86 on the patient level for revealing angiographically significant CAD (luminal stenosis ≥ 50%), with higher specificity when assessed on the vessel level (0.93) [32]. However, the diagnostic accuracy of wall motion abnormalities induced by dobutamine S-CMR is significantly influenced by LV geometry, with the lowest performance in patients with increased LV concentricity compared to those with normal geometry and eccentric hypertrophy (0.73 versus 0.87 versus 0.90, respectively) in detecting coronary stenosis ≥ 70% [47].

All these data have provided a defined role of S-CMR in the diagnosis of known or suspected CAD, with a high accuracy in relation to both coronary artery assessment and functional examinations, in particular SPECT.

## 4. Prognostic Role of Stress CMR

To allow appropriate management of CAD patients, a reliable prognostic assessment with information on patient outcome should be provided.

### 4.1. Myocardial Ischemia

Functional evidence of ischemia remains the major criterion for prognostically relevant CAD [48–50]. In 2011, a study by Krittayaphong et al. assessed the prognostic value of combined myocardial perfusion CMR and LGE, thus identifying myocardial ischemia as the strongest predictor for hard cardiac events and major adverse cardiac events (MACE) among patients with known or suspected CAD [51]. Buckert et al. in a large, consecutive, and thereby unselected population of patients presenting with stable angina pectoris reported how patients with reversible perfusion defects significantly showed more cardiac deaths (*p* < 0.0001) and nonfatal myocardial infarction (*p* = 0.001) than in the control group. Again, myocardial ischemia resulted as the strongest independent predictor for adverse events, with a high negative predictive value in the absence of a perfusion deficit [52]. Recently, Heitner et al., in a multicentre study involving 9151 patients followed up for up to 10 years, demonstrated that patients with positive perfusion S-CMR tests had significantly higher annual mortality rates, compared to those with normal tests. Additionally, there was a relevant improvement in predicting adverse events (*p* < 0.001) when positive perfusion S-CMR was included as a variable in Cox regression models [53].

Currently, the exact definition of ischemic burden and thresholds for initiating revascularization remains a subject of considerable interest, since the extent of ischemia was proved to be directly related to the number of subsequent CAD events [54]. Observational data indicated that medical therapy alone may be associated with a reduced risk of death compared with revascularization in patients with less extensive ischemia (<10% of the myocardium), while a more severe ischemia extent (≥10% of the myocardium) demonstrated a reduced risk of CAD and all-cause death with coronary revascularization compared with medical therapy [55]. A threshold of ≥10% ischemic myocardium was identified to define treatment effectiveness [56]. In 2017, Vincenti et al. performed a prospective study in 1024 patients with known or suspected CAD who were referred for perfusion CMR to detect myocardial ischemia. Data evidenced how an ischemia burden involving ≥1.5 ischemic segments was the strongest predictor of hard clinical events, and the authors concluded that patients with zero or 1 ischemic segment could be safely deferred to revascularization [57]. Concerning the ischemia extent, a technical advantage of S-CMR over SPECT relies on its higher spatial resolution (3 mm × 3 mm vs. 10 mm × 10 mm in-plane spatial resolution), which allows recording of smaller myocardial areas of hypoperfusion resulting in an even better diagnostic performance [58].

Recently, the MR-INFORM study has suggested S-CMR as a selection criterion for patients to be initiated to revascularization. This is a large, multicentre, randomized controlled clinical effectiveness trial that randomized 918 patients with suspected CAD to a myocardial perfusion CMR-based strategy or an FFR-based strategy. In the results, S-CMR was associated with a significantly higher reduction of invasive revascularization procedures than FFR (35.7% vs. 45.0%, *p* = 0.005), while the percentage of patients free from angina at 12 months did not differ significantly between the two groups (49.2% for S-CMR versus 43.8% for FFR, *p* = 0.21), thus representing noninferiority of S-CMR versus FFR in predicting MACE [59].

### 4.2. LGE and EF%

Another advantage of S-CMR is based on its ability to give complementary information on cardiac function and myocardial viability, eventually supported by advanced deep learning-based analysis methods [60], which could additionally provide prognostic information [61].

Low left ventricular ejection fraction (LVEF) has traditionally represented a marker of poor outcome in post-MI patients, with LVEF ≤ 35% denoting high-risk patients who require more aggressive management [62]. Moreover, the prognostic role of LGE in the field of CAD has also been extensively assessed [63]. In a retrospective, multicentre study by Kwong et al. in 2019, among 2349 patients with stable chest pain, the absence of LGE, as well as of myocardial ischemia, was related to a low incidence of cardiac events (<1%), reduced need for coronary revascularization (1-3%), and low spending on subsequent ischemia follow-up [64]. Among STEMI patients, early or deferred CMR provided equivalent and powerful stratification strategies for outcome prediction [65].

If large data support consistent and robust prognostic CAD stratification by adenosine S-CMR, fewer studies have investigated the usefulness of dipyridamole S-CMR for predicting adverse clinical events. In 2016, Pontone et al. demonstrated how dipyridamole stress CMR could predict adverse outcomes in 793 consecutive patients symptomatic for chest pain irrespectively of the amount of LGE, thus suggesting a relevant prognostic value of dipyridamole S-CMR by allowing the assessment of both key phases (perfusion and wall motion) of the ischemic cascade. Patients with normal dipyridamole S-CMR had a low annual hard event rate (1.8%) in comparison with patients with an abnormal perfusion defect alone (3.6%) or patients with perfusion defect plus WMA (9.4%) [66].

Strong evidence, therefore, is available demonstrating the value of S-CMR in excluding prognostically relevant ischemia, although it is an ideal test to exclude relevant disease in patients with known or suspected CAD.

## 5. Advanced Diagnostic and Prognostic Goals for Stress CMR

Since symptoms among patients with CAD are often not uniform and atypical, objective, thus reproducible, diagnostic tests are advisable to both confirm the diagnosis and assess the event risk.

### 5.1. Quantitative Perfusion

Commonly, the analysis of S-CMR image data is performed visually, and semiquantitative and quantitative perfusion techniques are mainly restricted to the research field [67], despite their potential clinical utility. Although quantitative approaches are more time-consuming, they provide very high accuracy in detecting segmental and global impaired myocardial perfusion and may help discriminate diagnosis in particular cases such as multivessel coronary disease, microvascular dysfunction, or suspicion of inadequate vasodilator response [68, 69].

Newer techniques allow direct quantification of the signal from the myocardium during first-pass perfusion and reflect the absolute value of myocardial blood flow in each pixel of the image data. Different approaches such as the Fermi model, uptake model, 1-compartment model, model-independent deconvolution method, and 2 model-independent methods have been proposed and have been shown to have similar diagnostic performance [70]. They would advantageously permit fully automated workflow, pixel-wise flow calculation, single-bolus contrast injection, and rapid processing, allowing an easier performable quantitative analysis [71, 72].

Only few studies provide comparative data among commonly used vasodilator agents regarding their hyperaemic effect. Vasu et al. determined the vasodilator power of each stress agent through both rest and stress myocardial blood flow (MBF) quantification [44]. In this analysis, regadenoson showed a higher stress MBF than adenosine and dipyridamole (3.58 vs. 2.81 vs. 2.78 ml/min/g, respectively, *p* < 0.001), with equivalent vasodilator effect to adenosine (37.8 vs. 36.6 *μ*l/sec/g, *p* = NS) but with a persistent higher effect than dipyridamole (37.8 vs. 32.6 *μ*l/sec/g, *p* = 0.03) when corrected for heart rate. Therefore, based on quantitative data, a comparable hyperaemic effect of all stress agents could not be fairly assumed. Indeed, most recently Kotecha et al. demonstrated how direct quantification of MBF itself in adenosine stress studies provides a more accurate evaluation of hyperaemia than traditional splenic switch-off and blood pressure response [73], thus encouraging further comparison among stress agents in larger and randomized studies through MBF and coronary flow quantification.

### 5.2. Mapping Sequences

Multiple studies have enhanced the increasing role of additional mapping sequences in CMR protocols, and potentially, the application of mapping sequences may help detect myocardial ischemia [74]. Stress native T1-mapping T2-mapping could potentially target changes in native myocardial T1 values under vasodilation stress (“T1 reactivity”), which reflect alterations in myocardial blood volume consequent to inducible ischemia [75, 76]. However, currently little evidence exists to allow an affordable use of these sequences in S-CMR protocols, which is still under research.

### 5.3. Feature-Tracking Analysis

**Figure 2:**
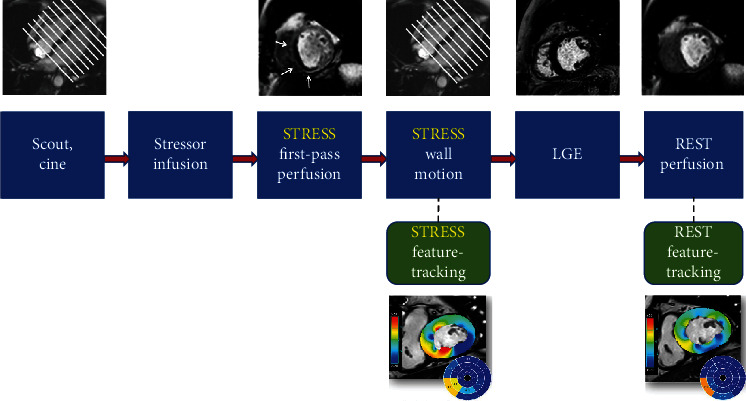
A potential protocol for stress cardiovascular magnetic resonance integrated with feature tracking analysis in both rest and stress phases.

Strain analysis has been lastly demonstrated to provide useful information on the presence of ischemia and on patient outcome in S-CMR studies. As widely evidenced, myocardial strain imaging allows quantification of subtle changes of LV function that typically precede a reduction in LVEF [77]. Garg et al. in 2018 provided the first evidence that a reduction in global longitudinal strain (GLS) at peak myocardial hyperaemic stress could be related to the presence of a perfusion defect in patients with suspected CAD [78]. Eventually a protocol of S-CMR may be integrated with strain analysis, without even significantly prolonging overall time acquisition, as shown in Figure 2.

Interestingly, Palmisano et al. evaluated both adenosine S-CMR mapping and strain data in 28 patients with refractory angina who underwent a coronary sinus Reducer implantation. After implantation, myocardial perfusion along with longitudinal (−16 to −19%; *p* = 0.0192) and circumferential strain (−18 to −21%; *p* = 0.0017) improved, without significant changes in radial, circumferential, and longitudinal strain rate (*p* > 0.05) and native T1 and extracellular volume (ECV) [79]. Advantages of both native mapping and feature tracking could relate to the possibility of achieving a sensitive, noninvasive, quantitative measure of myocardial ischemia and tissue alterations, even without the need for contrast agents, which is desirable due to increasing age and frequent concomitant renal disease in CAD patients. Poli et al. have tested the feasibility and reliability of noncontrast adenosine S-CMR T1 mapping in 58 patients under haemodialysis treatment and proved excellent test-retest reliability of rest and stress native T1 [80].

More scientific evidence is needed to prove the diagnostic performance and risk stratification power of such quantitative approaches. If these become more feasible and robust, they will potentially impact routine CAD management.

## 6. Current Challenges of Stress CMR

### 6.1. Main Limitations

A major challenge of S-CMR in clinical practice relies on its limited spatial coverage (only three short-axis slices) in perfusion studies, which may miss the presence of disease, compared to PET [81]. Perfusion S-CMR also has reduced applicability in patients with cardiac devices, which are increasingly prevalent, often due to large susceptibility artefacts that significantly impact image quality, especially in perfusion studies [82]. Moreover, S-CMR provides limited direct coronary stenosis analysis with reduced spatial resolution in comparison to CCTA. Nonlinear relationship between blood flow and tracer as well as between tracer and image signal has been proved, with contrast agent nonlinearity further affecting myocardial ischemia quantification [83].

Although S-CMR is commonly known as a safe technique due to its nonionizing effect, recent discussion has been raised on the potential acute effect on leucocyte DNA in 1.5 T CMR studies. In a recent paper by Critchley et al., CMR was demonstrated in both *in vivo* and *in vitro* studies not to cause DNA double-strand breaks and not to cause loss of leucocyte activity *in vitro*. However, CMR caused a relevant reduction of leucocytes viability *in vivo* [84]. All these results might raise suspicion for new detrimental effects of CMR, but larger clinical studies should be provided to prove the clinical impact of the described phenomenon.

### 6.2. Comparison with Other Imaging Modalities Assessing Myocardial Ischemia

The greatest applicability of CCTA in relation to myocardial ischemia is based on its high spatial resolution with precise evaluation of coronary stenosis and plaque characterization, as outlined in a recent Consensus Statement [85]. However, disadvantages of CCTA regard the use of ionizing radiation, limited temporal resolution, and low contrast-to-noise ratio, which may affect image quality together with beam and scatter artefacts [86].

PET represents the current technical standard for quantitative perfusion imaging, and recently introduced tracers and ^13^N-ammonia cyclotrons have improved its clinical applicability and cost-effectiveness [87]. Despite its technical appropriateness for assessment of myocardial ischemia, PET is currently limited by reduced availability and lower spatial resolution in comparison to S-CMR [88]. A hybrid PET-CT approach may allow a more comprehensive study of complex diseases, such as multivessel CAD [85]. In opposition, SPECT is widely available in clinical practice and represents the most frequently used modality for perfusion imaging. Due to recent technological evolution, such as the introduction of new dedicated cameras and compartment modelling, absolute values of MBF may be provided by SPECT with improved sensitivity, even in patients with high BMI, previously considered challenging [89]. However, SPECT is also an ionizing technique, with lower spatial resolution and lower image quality with need for attenuation and motion correction [90]. Stress echocardiography provides a rapid, widely available, nonionizing evaluation of myocardial ischemia, with potential for bedside applications. But despite its clinical usefulness, stress echocardiography is not applicable for coronary stenosis severity analysis and actually lacks in automated quantification of perfusion studies [91].

In conclusion, all these imaging modalities present different advantages and disadvantages for myocardial perfusion assessment, and the best clinical practice should be based on the choice of the most appropriate test for each clinical presentation, disease stage, and centre expertise and availability.

### 6.3. Future Perspectives

Current challenges and limitations of S-CMR will potentially be overcome in the next future thanks to technical evolutions involving both study acquisition and postprocessing phases, eventually leading to less time-consuming and more cost-effective studies.

For example, multitasking CMR has been defined in a study by Christodoulou et al. as a motion-resolved imaging modality with multitime dimensions that can adequately perform quantitative studies based on T1 and T2 relaxation constants without need for cardiac and/or breathing synchronization [92]. This may represent a future direction for CMR and possibly also for stress studies, by providing complete dataset of first-pass time-resolved native T1 mapping perfusion together with quantitative information on oedema and fibrosis within a single sequence.

Another appraisal should be outlined on the evolving role of deep-learning algorithms for fully automated quantification of CMR data [93], possibly supported by supervision with a rapid and high-quality confirmation of clinical images [94].

## 7. Conclusions

Accurate and informative diagnostic capability and prognostic relevance are essential requirements for patient management in the context of CAD, as underlined by the recent 2019 ESC guidelines on CCS. Among the available diagnostic modalities, S-CMR showed an overall high sensitivity and specificity for the detection of anatomically significant CAD (90% and 80%, respectively) and functionally significant CAD (89% and 87%, respectively). Appropriate selection of patients who undergo S-CMR potentially provides further strength to its diagnostic accuracy, which has been widely validated in a large body of evidence and more recently demonstrated clinical effectiveness in direct guiding revascularization in the presence of myocardial ischemia. Moreover, S-CMR achieves valuable prognostic information that ranges from the extent of myocardial ischemia itself and presence and transmurality of myocardial scarring to the entity of left ventricular remodelling and the impact on systolic function. Finally, future data on quantitative, objective, and sensitive parameters are expected to yield additional strength to S-CMR in a real-world setting, thus delivering measurements that are accurate and highly reproducible. Given its safety and multiparametric assessment both in terms of diagnosis and prognosis among CAD patients, S-CMR represents an invaluable modality for validating the efficacy of treatment as well as monitoring disease progress. However, despite being a powerful tool, more evidence is needed, especially for quantitative data, to directly translate S-CMR results into routine clinical practice and to provide greater feasibility of a customized patient-tailored approach.

## Figures and Tables

**Table 1:** Characteristics of defined studies regarding the diagnostic performance of stress perfusion cardiovascular magnetic resonance.

Author	Reference	*N*	Sensitivity (%)	Specificity (%)	Year
Schwitter et al. [31]	S-CMR vs. ICA	48	87%	85%	2001
Nandalur et al. [32]	S-CMR vs. ICA (meta-analysis)	1183	91%	81%	2007
Schwitter et al. [33]	S-CMR and SPECT vs. ICA	234	67%	85%	2008
Greenwood et al. [36]	S-CMR and SPECT vs. ICA	752	86%	83%	2012
Schwitter et al. [35]	S-CMR and SPECT vs. ICA	533	75%	59%	2013
Greenwood et al. [37]	S-CMR and SPECT vs. ICA	235	88%	83%	2014
Takx et al. [41]	S-CMR vs. FFR-ICA (meta-analysis)	798	89%	87%	2015
Danad et al. [38]	S-CMR vs. FFR-ICA (meta-analysis)	3788	90%	94%	2017
Pontone et al. [42]	S-CMR vs. FFR-ICA (meta-analysis)	1085	87%	88%	2019

S-CMR: stress cardiovascular magnetic resonance; ICA: invasive coronary angiography; SPECT: single-photon emission computed tomography; FFR-ICA: fractional flow reserve derived from invasive coronary angiography.

## Data Availability

The scientific data supporting this review article are from previously reported studies and datasets, which have been cited. The processed data are available from the corresponding author upon request.
